# Comparison of the Efficacy of Rosuvastatin 5 mg and 10 mg in Patients of Type 2 Diabetes Mellitus With Dyslipidemia

**DOI:** 10.7759/cureus.22595

**Published:** 2022-02-25

**Authors:** Mudassar Aleem, Alina Zainab, Azfar Hameed, Abdul Basit Khan, Syed Zahid Ali, Shifa Younus

**Affiliations:** 1 Internal Medicine, Nishtar Medical University, Multan, PAK; 2 Internal Medicine, Combined Military Hospital, Multan, PAK; 3 Neurology, Harvard Medical School, Boston, USA

**Keywords:** cholesterol, statins, type 2 diabetes mellitus, dyslipidemia, rosuvastatin

## Abstract

Objectives

We did this study intending to compare the efficacy of rosuvastatin 5 mg and 10 mg in patients of type 2 diabetes mellitus with dyslipidemia by validating their effect on lipid profile and the side effects.

Methodology

This study was carried out at the outpatient department of a tertiary care hospital in Multan. Three hundred patients of both genders were included. The research approach employed a parallel-controlled, randomized study. After taking relevant history and physical examination, each patient’s fasting venous blood samples were taken and sent to the institutional laboratory to analyze glycated hemoglobin (HbA1c), baseline lipid levels for cholesterol, triglycerides, low-density lipoprotein (LDL), very-low-density lipoprotein (VLDL), and high-density lipoprotein (HDL). Patients were divided into two groups based on the drug administered. One group was prescribed rosuvastatin 5 mg, and the other group was prescribed rosuvastatin 10 mg. Patients were followed up after six months to record the latest lipid profile. Data analysis was done through SPSS version 24.

Results

Patients in the two groups had similar lipid levels to start with. After six months of therapy, total serum cholesterol, triglycerides, and LDL-C were reduced to statistically significant levels in group two compared to group one. However, both groups showed a similar increase in serum levels of HDL-C. Patients treated with 10 mg rosuvastatin showed a slight decrease in BMI. Nine patients treated with 10 mg rosuvastatin reported myalgias compared to only one patient treated with a dose of 5 mg (p<0.005).

Conclusion

Our study concludes that both 5 mg and 10 mg of rosuvastatin exhibit the antihyperlipidemic effect, but high doses are associated with more side effects. Therefore, physicians should be aware of dose titration related to statins as it will ultimately lead to reduced cardiovascular mortality.

## Introduction

Dyslipidemia is a well-studied risk factor for developing diseases associated with atherosclerosis, including coronary heart disease (CHD) and ischemic stroke. There is abundant evidence suggesting that lowering low-density lipoprotein cholesterol (LDL-C) reduces the risk of cardiovascular diseases (CVDs) [1-2]. Both European and USA guidelines for CVD prevention recommend using 3-hydroxy-3-methylglutaryl coenzyme A reductase inhibitors (statins) as first-line therapy for dyslipidemia [3]. More recently, a National Cholesterol Education Program (NCEP) report has proposed to lower target levels to even more aggressive LDL-C goals for very high-risk patients [4].

Not only is the treatment of dyslipidemia associated with significantly improved outcomes in patients with these diseases, but also lipid-lowering is the most effective intervention in primary prevention [5]. Statins are the primary agents for managing dyslipidemia. In addition to the arithmetic reduction in lipid profile, they significantly reduce micro and macro-vascular events and all-cause mortality through their multitrait effects. It is well established that statins have antioxidant, anti-inflammatory effects, and antithrombotic properties that add to their clinical utility [6]. They also lead to improved endothelial dysfunction and a reduction in the growth of atherosclerotic plaque [7].

All existing statins have minor differences in pharmacokinetics and pharmacodynamics and hence in clinical efficacy and side effects profile [8]. Evidence from the current literature suggests that rosuvastatin causes a significant reduction in LDL-C and has a maximum rate of achieving therapeutic milestones than other statins [9]. However, such data from our country is restricted. It is well established that Asians may have a varied response from whites because of genetic differences in drug metabolism at the hepatic enzyme and drug transporter level depending on drug potency [10].

We did this study intending to compare the efficacy of rosuvastatin 5 mg with 10 mg in patients with type 2 diabetes mellitus with dyslipidemia. The results of this study might prove helpful, giving us clues about dosing and potential side effects of two different doses of rosuvastatin.

## Materials and methods

We conducted this prospective study at the outpatient department of a tertiary care hospital in Multan. Three hundred patients of both genders were included. Informed consent was taken from all of the patients involved. The research approach employed a parallel-controlled, randomized study to compare the efficacy of rosuvastatin 5 mg and 10 mg in patients with type 2 diabetes mellitus.

Inclusion criteria

Type 2 diabetic patients with a previous history of myocardial infarction (MI) or stroke were included. The age ranged from 30 to 65 years.

Exclusion criteria

Those excluded from the study were the patients having liver disease, kidney disease, hypothyroidism, or any other acute or chronic disease. In addition, patients taking other lipid-lowering drugs, patients with type 1 diabetes, and those using systemic or inhaled glucocorticoids or other medication known to interfere with lipid metabolism were also excluded.

Data collection procedure

After approval from the institutional review board, this study was done in the outpatient department of medicine in Nishtar Hospital, Multan. After taking relevant history and physical examination, each patient’s fasting venous blood samples were taken and sent to the institutional laboratory to analyze glycated hemoglobin (HbA1c), baseline lipid levels for cholesterol, triglycerides, LDL, very-low-density lipoprotein (VLDL), and high-density lipoprotein (HDL). The BMI of the patients, along with the waist circumference, was calculated in the outpatient facility. Patients were divided into two groups based on the drug administered. One group was prescribed rosuvastatin 5 mg, and the other was prescribed rosuvastatin 10 mg. A specialized proforma was designed to handle all the study information. Patients were be followed up after six months to record the latest lipid profile.

Data analysis

Data analysis was done through SPSS version 24 (IBM Corp, Armonk, NY, United States). The data was reported as means ± standard error. Statistical comparisons were made using independent 𝑡-tests for values before and after the six-month treatment, paired 𝑡-tests between groups, and 𝑃 values < 0.05 were considered statistically significant. Conclusions were made accordingly.

## Results

All patients completed the study. The sociodemographic details and the clinical baseline investigation values of the patients of both groups are given in Table 1.

**Table 1:** Sociodemographic details and the clinical baseline investigation values of the patients of both groups (n=300) HbA1C: glycated hemoglobin

Patient’ characteristics	Rosuvastatin 5 mg group (N=150)	Rosuvastatin 10 mg group (n=150)
Age, years (mean ± SD)	53.7±3.2	54.4±2.8
Gender (%)		
Male	118(79)	127(85)
Female	32(21)	33(15)
Body mass index (BMI), kg/m² (mean ± SD)	28.13±2.6	29.84±2.8
Waist circumference, cm (mean ± SD)	96.2±6.7	99.4±7.2
Systolic blood pressure, mmHg (mean ± SD)	133.2±4.7	136.4±5.3
HbA1C (%) (mean ± SD)	8.3± 0.67	9.1±1.1
Serum total cholesterol, mg/dl (mean ± SD)	204.32±18.1	208.1±22.4
Serum triglycerides, mg/dl (mean ± SD)	165.73±23.6	161.21±25.7
Serum low-density lipoproteins (LDL), mg/dl (mean ± SD)	144.15±20.4	147.4±24.1
Serum high-density lipoproteins (HDL), mg/dl (mean ± SD)	27.7±5.4	31.2±4.5

Patients in the two groups had similar lipid levels to start with. After six months of therapy, total serum cholesterol, triglycerides, and LDL-C were reduced to statistically significant levels in group two compared to group one. However, both groups showed a similar increase in the serum levels of HDL-C. The absolute changes in the baseline levels of the lipid profile are shown in Table 2. Nine patients treated with 10 mg rosuvastatin reported myalgias compared to only one patient treated with a dose of 5 mg (*P*<0.005).

**Table 2:** Absolute changes in the baseline values of each parameter of lipid profile LDL-C: low-density lipoprotein cholesterol; HDL-C: high-density lipoprotein cholesterol

Variables	Rosuvastatin 5 mg	Rosuvastatin 10 mg	*P*-value
Serum total cholesterol (mg/dl) (mean ± SD)	147.2±14.5	131.6±12.1	0.031
Serum triglycerides (mg/dl) (mean ± SD)	98.4±9.4	81.7±7.2	0.028
Serum low-density lipoprotein cholesterol (LDL-C) (mg/dl) (mean ± SD)	85.5±6.8	72.4±5.1	0.041
Serum high-density lipoprotein cholesterol (HDL-C) (mg/dl) (mean ± SD)	44.5±3.3	45.1±4.7	0.87

**Figure 1:**
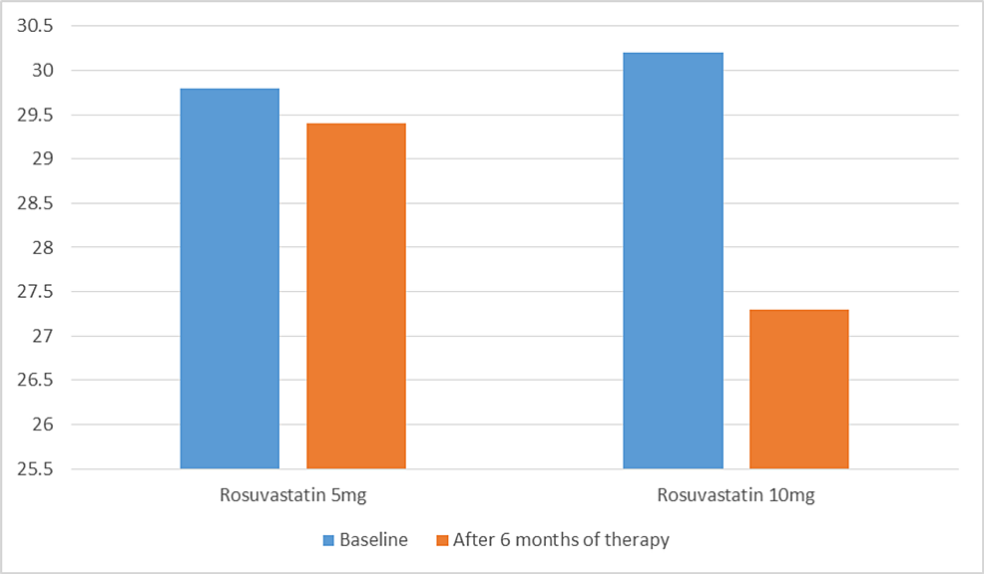
Body mass index (BMI) of patients with type 2 diabetes before and after six-month treatment of rosuvastatin 5 mg or 10 mg

After six months of therapy, no statistically significant difference was observed in comparing the BMI profile of both groups. However, patients treated with 10 mg rosuvastatin showed a slight decrease in BMI (29.84±2.8 kg/m^2^ versus 27.7±1.2 kg/m^2^). Patients in group one did not show any statistically significant reduction in BMI (Figure 1).

## Discussion

Our study reported that both 5 mg and 10 mg rosuvastatin significantly decreases blood cholesterol, triglycerides, and LDL levels. The decline showed by group two, which was treated with 10 mg rosuvastatin, was statistically significantly higher than the other group. Both groups showed an insignificant rise in the levels of HDL. Both antihyperlipidemic regimens were generally well-tolerated, and there was no significant rise in the liver or muscle enzyme levels. A few patients taking 10 mg rosuvastatin reported mild to intermediate myalgia, which was resolved with drug cessation and supportive therapy. Free fatty acids (FFAs) constitute a significant energy source derived from adipose tissue during the breakdown of triglycerides. Dyslipidemia is closely related to cardiometabolic risk factors, including metabolic syndrome, oxidative stress, atherosclerosis, and cardiovascular events [11-14]. A study conducted by Pilz et al. showed that FFA level was an independent predictor of all-cause and cardiovascular mortality [15]. In our study, the patients showed a significant reduction in LDL levels with rosuvastatin. This finding parallels existing literature, the most popular amongst them is the STELLAR trial [16].

The clinicians should keep in mind the doses of different statins, especially rosuvastatin, while applying the results of this study to clinical practice. Dose adjustment of stain is essential as other statins can cause an equivalent reduction in the levels of LDL [17]. Therefore, choosing a statin that is more effective at initial doses will minimize the need for dose titration and facilitate goal achievement, ultimately leading to benefits in cardiovascular risk reduction. HDL plays a protective role against the growth of atherosclerotic plaques, especially in the coronary vessels, and a reduced level of HDL is an isolated risk factor for cardiovascular morbidity and mortality [18]. In our study, there was a rise in the HDL level of both groups, but the increment in the group of patients taking 10 mg rosuvastatin was statistically significant.

Both 5 mg and 10 mg rosuvastatin were well tolerated in our study. Few patients in the 10 mg rosuvastatin group reported mild to intermediate body aches, but the laboratory workup failed to show any significant myalgia or derangement in hepatic enzyme levels. However, we could not monitor the laboratory workup due to a lack of funding and frequent follow-up. Our study included a small cohort of patients compared to already existing studies. Large trials including a much larger patient population should be conducted to compare the efficacy and side effects of different doses of statins.

## Conclusions

Our study concludes that both 5 mg and 10 mg of rosuvastatin exhibit the antihyperlipidemic effect. The patients treated with 10 mg rosuvastatin showed more reduction in lipid levels and reported more side effects. Therefore, physicians should be aware of dose titration related to statins as it will ultimately lead to reduced cardiovascular mortality.
